# Can Field-Based Screening Predict ACL Injury Risk in Women Footballers? External Validation of a Prediction Model

**DOI:** 10.1177/19417381261457613

**Published:** 2026-07-23

**Authors:** Yuri Lopes Lima, Tyler Collings, Michelle Hall, Laura E. Diamond, Matthew N. Bourne

**Affiliations:** † School of Allied Health, Sport and Social Work, Griffith University, Gold Coast, Australia; ‡ Australian Centre for Precision Health and Technology (PRECISE), Griffith University, Gold Coast, Australia; § Musculoskeletal Research Hub, Charles Perkins Centre, Sydney School of Health Sciences, Faculty of Medicine and Health, The University of Sydney, Sydney, Australia

**Keywords:** female, knee, movement, prevention

## Abstract

**Background:**

Anterior cruciate ligament (ACL) rupture is a devastating injury that occurs 3 to 7 times more frequently in women footballers than in their male counterparts. Identifying players at elevated risk is critical for targeted injury prevention; however, no ACL injury prediction model has undergone external validation in women. Therefore, this study aimed to externally validate a field-based ACL injury prediction model in women footballers.

**Hypothesis:**

A previously developed field-based ACL injury prediction model would demonstrate good predictive performance in an independent cohort of women footballers.

**Study Design:**

Prospective cohort study.

**Level of Evidence:**

Level 2.

**Methods:**

Women footballers (*n* = 320) completed preseason assessments of single-leg hop kinematics, countermovement jump (CMJ) kinetics, hip adductor/abductor strength, and self-reported injury history. Players were followed prospectively for 18 months for noncontact ACL injuries. Model performance was evaluated via discrimination (area under the curve [AUC]) and calibration (observed versus predicted risk). The model was updated subsequently using the combined development and validation cohorts (*n* = 642), incorporating the hip adductor/abductor strength ratio and ipsilateral trunk flexion angles. Internal validation was performed via bootstrapping.

**Results:**

In the external validation cohort, the original model demonstrated reduced discrimination (AUC, 0.67; 95% CI, 0.49-0.82) and poor calibration (intercept, –0.79; slope, 0.53), indicating risk overestimation. The updated 5-variable model improved discrimination (apparent AUC, 0.76; optimism-corrected AUC, 0.72; 95% CI, 0.61-0.82), and calibration intercept (–0.00), but the calibration slope (1.38) and instability across bootstrapped samples indicated imprecise individual risk estimates.

**Conclusion:**

The original ACL injury prediction model did not generalize well to an independent cohort of women footballers. Although model updating improved overall predictive performance, calibration remained suboptimal, and predictions were unstable. Field-based strength and biomechanical assessments may support group-level ACL injury risk stratification but are not yet suitable for precise individual risk prediction.

**Clinical Relevance:**

Field-based measures of strength and biomechanics can help identify groups of women footballers at elevated ACL injury risk, achieving 72% classification accuracy. These screening measures are practical and scalable, requiring <10 minutes per player, and assess modifiable factors that may inform targeted injury prevention strategies.

Participation of women and girls in Australian football and soccer has grown significantly in recent years.^22^ However, women experience disproportionately high rates of lower limb injury,^22^ with anterior cruciate ligament (ACL) injuries among the most catastrophic and costly.^42^ Women and girls are 3 to 7 times more likely to sustain an ACL injury than their male counterparts.^50^ Alarmingly, 1 in 3 players fail to return to play after ACL injury,^7^ and up to 22% experience reinjury.^48^

Understanding the factors that increase a player’s risk of injury represents an important step towards developing targeted injury prevention interventions.^21^ Most ACL injuries occur without contact (or via indirect contact) when changing direction or landing from a jump.^10^ Consequently, a large body of work has examined biomechanical profiles,^17^ and the predictive associations between biomechanical variables and subsequent injury. Lower hip and knee flexion angles,^24,30,31^ greater knee abduction angles and moments,^12,18,24,29^ and high ground reaction forces (GRFs) during change of direction and landing,^24,31^ have been associated with future noncontact (i.e., without direct impact from another person or object) ACL injury, albeit inconsistently.^11,14,27^ However, most prospective studies have examined potential risk factors in isolation (i.e., using univariable analysis) without considering a model integrating multiple factors to estimate the probability of injury (multivariable prediction models),^44^ limiting their predictive utility.

Prediction models integrate data from multiple predictors to estimate a person’s future injury risk and, when rigorously developed and validated, can support clinicians in informed decision-making.^8^ However, most sports injury prediction models are characterized by a high risk of bias (98%), small sample sizes, and a lack of external validation.^9^ This highlights the need for improved methodological rigor, larger and more representative samples through multicenter collaboration, and robust external validation of existing models to facilitate their safe and effective translation into clinical practice.

Our previous work addressed this gap by developing the first field-based multivariable prediction model for ACL injury in women/girl footballers.^12^ In a cohort of 322 elite players, a model including ACL injury history, peak take-off force during a countermovement jump (CMJ), and dynamic knee valgus during hopping correctly classified 78% of subsequently injured players.^12^ However, external validation in an independent cohort is required to assess the generalizability of the model before clinical application.^8,35^

In addition, two other field-based variables not included in our original model - hip adductor/abductor strength ratio and ipsilateral trunk flexion angles during hopping - were independently associated with ACL injury.^12^ These variables influence frontal plane knee control and loading,^19,38^ are measured easily in field-based settings, and may enhance model performance if incorporated.

The primary aim of this study was to externally validate the original field-based ACL injury prediction model in an independent cohort of women and girl footballers. A secondary aim was to update the model by incorporating additional predictors if external validation indicated suboptimal performance.

## Methods

This study followed the PROGnosis RESearch Strategy (PROGRESS) framework,^44^ and is reported according to the Transparent Reporting of multivariable prediction model for Individual Prognosis or Diagnosis (TRIPOD) guidelines.^35^

### Sample Size Calculation

We estimated the sample size for the validation dataset based on the area under the receiver operating curve (AUC) of 0.78 of the development model.^12^ We aimed for a standard error of the AUC of 0.06, which corresponds to a CI width of 0.24 (i.e., 0.66 to 0.90). With an ACL injury outcome proportion estimated at 5.4%, a sample size of 290 participants was required.^40^ We anticipated to have 10% of participants with missing outcomes or predictors; therefore, we increased the required sample size to 320 to account for missing data.^41^

### Source of Data

Australian Rules football players were recruited via team staff from clubs in Queensland, New South Wales, and Victoria. Soccer players were recruited from all around Australia for the development cohort, and from a Queensland state sports academy for the validation cohort.

### Participants

Eligibility criteria included: woman/girl >16 years of age; currently training or competing for a state-level or professional football club in Australian Rules football or soccer; no current injuries that may impact the participant’s ability to perform the tests; and not pregnant. All players provided written, informed consent to participate. Players <18 years of age sought permission and a signature from a parent, guardian, or coach. The study was approved by the University’s ethics board (GU: 2019/423).

The original model was developed on 322 women/girls Australian Rules football and soccer players (277 players with complete outcome follow-up data were used to develop the original model) with baseline testing occurring in preseason between 2019 and 2021.^12^ The validation cohort consisted of 320 women/girls Australian Rules football and soccer players with baseline testing in the 2023/2024 season. The injury follow-up for both cohorts was 18 months.

### Outcomes

The outcome was a complete ACL rupture due to a noncontact mechanism. Injury details (injured side, date of injury, and place (e.g., training/game)) were reported at the end of the follow-up period by team medical staff via a standardized report form (Supplementary Material 1, available in the online version of this article). ^23^ The diagnosis of ACL rupture was made by the team physiotherapists/physicians with or without confirmation through imaging tests.

### Field-Based Testing Battery and Predictor Variables

Demographics (age, height, limb dominance [kicking foot], history of ACL injury [i.e., complete ACL rupture in either leg]) were collected at baseline through a standardized questionnaire (Supplementary Material 2, available online). Subsequently, players underwent assessments of double-leg CMJ kinetics, trunk, and lower limb kinematics during a single-leg triple hop test, and isometric hip adduction/abduction strength at a single timepoint in preseason. The tests were conducted by 1 to 2 researchers, taking no longer than 10 minutes per player.

#### Double-leg CMJ

Double-leg CMJs were performed using portable dual force plates (ForceDecks, Vald Performance Pty Ltd). While standing upright with 1 foot in each force plate and the hands on the hips, participants were instructed to perform a quick countermovement to a self-selected depth and jump as high as possible. A trial was considered valid if participants kept their hands on their hips at all times and landed on both feet, in each assigned force plate. Players performed 3 trials, each separated by 5-10 seconds rest.

Peak take-off force was extracted from each of the 3 trials for the left and right legs and the mean of 3 trials was used for analysis. Peak take-off force was defined as the peak force over the concentric/eccentric phase of the jump phase. Peak force was normalized to body weight (BW) by dividing the GRF in each leg by the total weight force in Newtons (N). The ForceDecks peak take-off force has good to excellent validity (4 N difference) compared to laboratory force plates and excellent reliability (intraclass correlation coefficient [ICC] = 0.99).^13^

#### Single-Leg Triple Vertical Jump

Single-leg triple vertical jump kinematics were assessed using a markerless motion capture system (HumanTrak, Vald Performance Pty Ltd). The HumanTrak system consists of a Microsoft Azure Kinect camera containing red-green-blue cameras to record motion and infrared cameras that determine depth. The Microsoft body tracking software developer kit was then used to predict 3-dimensional (3-D) coordinates of key points (n = 21) located at the foot, ankle, knee, hip, spine, shoulder, elbow, wrist, neck, and head. Key points located at joint centers were used to define segment orientations and calculate joint angles. To perform the single-leg triple vertical jump, participants were positioned approximately 2.5 meters in front of the camera. Standing on 1 foot, and hands on their hips, participants performed 3 consecutive hops for maximum height in a continuous motion. Participants received standardized encouragement to jump as high as they could. A trial was first performed on each leg to warm up and practice the task. Peak dynamic knee valgus and ipsilateral trunk flexion angles were extracted from the landing phase, defined as the phase between estimated first ground contact (center of mass below standing height) and the lowest position of the center of mass. The dynamic knee valgus angle (2-dimensional, frontal plane projection angle) was calculated as the angle in degrees between the femur segment (hip to knee keypoint) and the shank segment (knee to ankle keypoint). Three trials were performed on each leg, for a total of 9 hops on each leg (3 trials times 3 hops). All trials were analyzed by taking the mean of the 9 landings. The HumanTrak system has moderate reliability for dynamic knee valgus (ICC = 0.65) and lateral trunk flexion (ICC = 0.58).^12^

#### Hip Adductor and Abductor Isometric Strength

Isometric strength of the hip adductors and abductors was measured using a portable fixed frame dynamometer (ForceFrame, Vald Performance). The ForceFrame consists of uniaxial force transducers embedded in outer and inner pads attached to a fixed frame. The ForceFrame has good to excellent reliability (ICC = 0.86-0.92) for isometric hip adduction and abduction strength.^28^ Players laid in a supine position, with knee extended, and malleoli aligned to the pads. Players performed 3 maximal voluntary isometric contractions alternating between abduction and adduction. Each trial was held for 5 seconds with a 5-second rest interval. The maximum adductor and abductor force during the 3 trials and the ratio of the maximum adductor to abductor force were used for analysis.

### Missing Data

Reasons for missing predictor data included equipment malfunction and equipment availability (missing completely at random; i.e., peak take-off force and dynamic knee valgus), omitted responses to questions about previous injury (missing at random), and the nature of the question itself (missing not at random; i.e., contraceptive use). A sensitivity analysis using a complete-case analysis assessed potential bias due to missing data.

### Statistical Analysis

#### Univariable Risk Factors

Standardized odds ratios (OR) were calculated for continuous predictors, representing the change in odds of ACL injury per 1 standard deviation increase. For binary predictors, ORs were estimated by comparing the odds of the outcome between players with and without the characteristic of interest.^6^ An OR >1 indicates increased odds of sustaining an ACL injury, whereas an OR <1 indicates a decrease.^16^ OR was considered statistically significant if the Wald 95% CIs did not include 1. For tests in which a single limb was treated as the unit of analysis (e.g., dynamic knee valgus, peak take-off force, hip adduction/abduction ratio), values from the injured limb were used for players who sustained an ACL injury, whereas the mean of both limbs was used for players who did not sustain an injury.

#### Prediction Model External Validation

Predictive performance of the original ACL injury prediction model (previous ACL injury, peak take-off force, and dynamic knee valgus) was assessed by discrimination and calibration on the external validation cohort. Discrimination is the ability of a model to differentiate between the athletes who did and did not sustain a subsequent ACL injury.^45^ Discrimination was quantified by the AUC, where 0.5 represents discrimination no better than chance and 1.0 represents perfect discrimination.^37^ Values between 0.60 and 0.75 were interpreted as possibly helpful, and >0.75 as useful.^1^ Calibration refers to the agreement between the predicted and observed risks of sustaining an outcome.^47^ Calibration was assessed by plotting observed risk versus predicted risk and estimating the calibration curve with an intercept of zero and a slope of 1 indicating perfect calibration.^46^

#### Prediction Model Update

**Figure 1.**
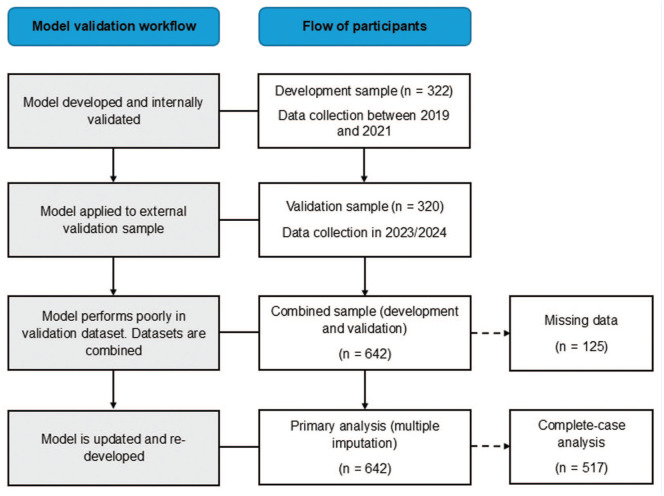
Model validation workflow and flow of participants.

To improve the model’s performance, we added hip adductor/abductor strength ratio and trunk ipsilateral flexion to the model using both development and validation cohorts. Apparent AUC of the updated model was calculated by assessing its performance in the combined cohort. Missing predictor values were imputed using predictive mean matching. A total of 20 different datasets were created as part of the multiple imputation approach. The final model was derived by averaging the regression coefficients across the multiple imputed datasets using Rubin’s rules and Ridge regression.^2^ Internal validation of the updated model was performed through bootstrapping to obtain optimism corrected AUC. First, a total of 1000 bootstrap samples of the same size as the combined cohort were drawn with replacement. Predictive mean matching (*m* = 20) was used to create imputed datasets to fit a model to each bootstrap sample. Next, apparent AUC for each bootstrap model was computed. Each bootstrap model was applied to the combined cohort sample and AUC was calculated. Then, optimism was calculated for each bootstrap sample by subtracting the AUC of the bootstrapped model applied to the combined cohort to the AUC of bootstrap model applied to bootstrap sample. All the optimism values were averaged to estimate overall optimism. Finally, the optimism-corrected AUC was calculated by subtracting the overall optimism from the apparent AUC.^20^ Instability of the updated model predictions was assessed by plotting the calibration curves of the bootstrapped models assessed in the combined cohort. Instability quantifies how different the model would be if developed in a different sample of the same size in the same population.^39^ Model validation workflow is shown in Figure 1. All statistical analyses were performed using RStudio (Version 2022.07.2) using the packages rms, mice, pROC, glmnet, and CalibrationCurves.

## Results

### Injury Characteristics

In the development cohort, 15 of 322 players (4.6%) sustained a noncontact ACL rupture. Of these, 5 were reinjuries (4 were contralateral injuries) and 10 (66.6%) occurred in the nondominant leg. In the validation cohort, 10 of 320 players (3.1%) sustained a noncontact ACL rupture, of which 9 were index injuries, with 6 (60%) occurring in the nondominant leg. In the combined cohort (*n* = 642), 25 players (3.9%) sustained an ACL injury; 19 were index injuries and 6 were reinjuries (5 in the contralateral leg). Of the 25 total injuries, 16 occurred in the nondominant leg (Table 1).

**Table 1.** Demographics and predictor values for the development and validation cohorts

	Development cohort					Validation cohort				
	Injured (n ≤ 15)	Uninjured (n ≤ 307)	Total (n ≤3 22)	Missing values, n (%)	Standardized OR (95% CI)	Injured (n ≤1 0)	Uninjured (n ≤ 310)	Total (n ≤ 320)	Missing values, n (%)	Standardized OR (95% CI)
Age, years^a^	20 (4)	20 (9)	20 (8.7)	0 (0)	0.78 (0.44, 1.38)	20 (8.2)	22 (8)	22 (8)	2 (0.6)	0.90 (0.47, 1.74)
Height, m	1.76 (0.06)	1.77 (0.07)	1.77 (0.07)	64 (19.9)	0.90 (0.53, 1.54)	1.69 (0.06)	1.69 (0.08)	1.69 (0.08)	23 (7.1)	0.99 (0.51, 1.94)
BW, kg	65.3 (5.9)	65.0 (8.7)	65.0 (8.6)	36 (11.2)	1.05 (0.62, 1.78)	69.3 (10.9)	67.5 (10.3)	67.6 (10.3)	17 (5.3)	1.18 (0.65, 2.13)
Contraceptive use, n (%)^b^	4 (27)	64 (21)	68 (21)	0 (0)	1.38 (0.43, 4.48)	1 (11.1%)	68 (24.1%)	69 (24%)	30 (9.4)	0.39 (0.05, 3.19)
Previous ACL rupture, n (%)^b^	5 (33)	16 (6)	21 (7)	21 (6.5)	8.44 (2.58, 27.63)*	1 (10.0%)	36 (11.3%)	36 (11%)	0 (0)	0.87 (0.11, 7.1)
Previous knee sprain, n (%)^b^	2 (13)	19 (7)	21 (7)	21 (6.5)	2.16 (0.45, 10.29)	1 (10.0%)	23 (8.1%)	24 (8%)	28 (8.7)	1.25 (0.15, 10.32)
Previous hamstring strain, n (%)^b^	2 (13)	23 (8)	25 (8)	21 (6.5)	1.76 (0.37, 8.28)	2 (20.0%)	41 (14.5%)	43 (15%)	28 (8.7)	1.47 (0.30, 7.17)
Previous hip and groin pain, n (%)^b^	2 (13)	25 (9)	27 (9)	21 (6.5)	1.61 (0.34, 7.52)	3 (30.0%)	54 (19.2%)	57 (19%)	29 (9)	1.80 (0.45, 7.19)
CMJ peak take-off force, BW	1.23 (0.12)	1.15 (0.12)	1.15 (0.12)	36 (11.2)	1.77 (1.12, 2.82)*	1.23 (0.15)	1.17 (0.15)	1.17 (0.14)	17 (5.3)	1.44 (0.80, 2.56)
Maximum isometric hip abductor force, N	145 (22)	143 (24)	143 (24)	15 (4.6)	1.08 (0.65, 1.79)	151 (40)	140 (27)	140 (26)	21 (6.6)	1.47 (0.77, 2.80)
Maximum isometric hip adductor force, N	133 (26)	144 (27)	143 (27)	16 (5)	0.62 (0.35, 1.11)	139 (43)	134 (30)	135 (31)	14 (4.4)	1.18 (0.62, 2.24)
Hip adductor/abductor strength ratio	0.92 (0.17)	1.01 (0.14)	1.01 (0.14)	16 (5)	0.50 (0.28, 0.90)*	0.94 (0.24)	0.97 (0.16)	0.97 (0.16)	25 (7.8)	0.85 (0.44, 1.67)
Dynamic knee valgus, positive deg/varus negative deg	1.5 (5.8)	-2.1 (7.2)	-2.2 (7.1)	52 (16.1)	1.76 (1.00, 3.16)	3.1 (4.5)	-0.2 (3.6)	-0.2 (3.6)	15 (4.7)	2.59 (1.31, 5.12)*
Ipsilateral trunk flexion, positive deg	8.7 (3.9)	7.8 (2.2)	7.9 (2.3)	52 (16.1)	1.41 (0.88, 2.26)	10.9 (5.1)	8.6 (3.2)	8.7 (3.3)	15 (4.7)	1.78 (1.04, 3.04)*

Except for age and categorical variables, all variables are distributed normally and presented as mean (SD). ACL, anterior cruciate ligament; BW, bodyweight; CMJ, countermovement jump; OR, odds ratio.

a Group data presented as median (interquartile range).

b Group data presented as count (percentage).

* Statistically significant results at *P* < 0.05.

### Univariable Risk Factor Analysis

In the combined cohort, previous ACL rupture (OR, 3.37; 95% CI, 1.29, 8.83), higher CMJ peak take-off force (OR, 1.57; 95% CI, 1.10, 2.26), higher dynamic knee valgus (OR, 1.95; 95% CI, 1.24, 3.06), and higher ipsilateral trunk flexion (OR, 1.48; 95% CI, 1.05, 2.10) increased the odds of sustaining an ACL injury (Table 2).

**Table 2.** Demographics and predictor values for the combined cohort

	Combined cohort					
	Injured (n ≤ 25)	Uninjured (n ≤ 617)	Total (n ≤ 642)	Missing values, *n* (%)	*P* value (difference between injured and uninjured)	Standardized OR (95% CI)
Age, years^a^	20 (4)	21 (9)	21 (9)	2 (0.3)	0.530	0.81 (0.52, 1.25)
Height, m	1.74 (0.07)	1.73 (0.08)	1.73 (0.08)	87 (13.5)	0.659	1.09 (0.71, 1.65)
BW, kg	67.0 (8.4)	66.3 (9.6)	66.3 (9.6)	53 (8.2)	0.675	1.08 (0.73, 1.60)
Contraceptive use, n (%)^b^	5 (21)	132 (22)	137 (22)	30 (4.7)	1.000	0.91 (0.33, 2.48)
Previous ACL rupture, n (%)^b^	6 (24)	51 (9)	57 (9)	21 (3.3)	0.020*	3.37 (1.29, 8.83)*
Previous knee sprain, n (%)^b^	3 (12)	42 (7)	45 (8)	49 (7.6)	0.426	1.71 (0.49, 5.94)
Previous hamstring strain, n (%)^b^	4 (16)	64 (11)	68 (11)	49 (7.6)	0.514	1.50 (0.50, 4.51)
Previous hip and groin pain, n (%)^b^	5 (20)	79 (14)	84 (14)	50 (7.8)	0.380	1.54 (0.56, 4.23)
CMJ peak take-off force, BW	1.23 (0.13)	1.16 (0.14)	1.16 (0.13)	53 (8.2)	0.015*	1.57 (1.10, 2.26)*
Maximum isometric hip abductor force, N	147 (29)	142 (25)	142 (25)	36 (5.6)	0.368	1.24 (0.83, 1.84)
Maximum isometric hip adductor force, N	135 (33)	139 (29)	139 (29)	30 (4.7)	0.568	0.87 (0.57, 1.32)
Hip adductor/abductor strength ratio	0.93 (0.19)	0.99 (0.15)	0.99 (0.15)	41 (6.4)	0.141	0.67 (0.44, 1.02)
Dynamic knee valgus, positive deg/varus negative deg	2.1 (5.3)	-1.1 (5.6)	-1.1 (5.6)	67 (10.4)	0.005*	1.95 (1.24, 3.06)*
Ipsilateral trunk flexion, positive deg	9.6 (4.4)	8.3 (2.8)	8.3 (2.9)	67 (10.4)	0.145	1.48 (1.05, 2.10)*

Except for age and categorical variables, all variables are distributed normally and presented as mean (SD) and comparison between injured and uninjured performed by independent 2-tail *t-*test. ACL, anterior cruciate ligament; BW, body weight; CMJ, countermovement jump; N, Newton; OR, odds ratio.

a Comparison between injured and uninjured performed by Wilcoxon rank test and presented as median (interquartile range).

b Comparison between injured and uninjured compared with Fisher’s exact test for categorical variables and presented as count (%).

* Statistically significant results at *P* < 0.05.

### Prediction Model External Validation

**Figure 2.**
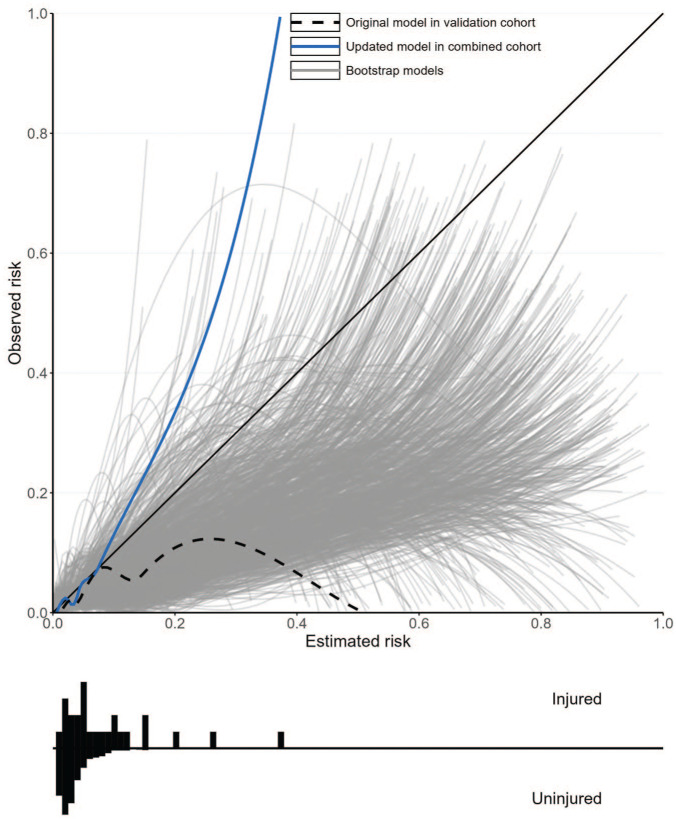
Calibration curve of the original model in the validation cohort (dashed line), updated model in the combined cohort (solid blue line), and calibration instability plot based on 1000 bootstrap samples (solid gray lines) and distribution of the predicted probabilities of the injured and uninjured players.

The original model demonstrated useful optimism-corrected performance in the development cohort (AUC, 0.78; 95% CI, 0.66, 0.90); however, its performance declined when applied to the validation cohort (AUC, 0.67; 95% CI, 0.49, 0.82) (Table 3). In addition, the model displayed miscalibration in the validation cohort, with a calibration intercept of –0.79 and slope of 0.53 indicating that predicted risks were overestimated and the model predictions were overly extreme (Figure 2).

**Table 3.** Predictor coefficients and performance for the original and updated models

Predictors	Original model (development cohort)	Original model (validation cohort)	Updated model (combined cohort)
Beta coefficients			
Intercept	−7.26	−7.26	−5.75
Previous ACL injury, yes	2.03	2.03	0.93
CMJ peak take-off force, BW	3.46	3.46	2.86
Dynamic knee valgus, deg	0.09	0.09	0.10
Hip adductor/abductor force ratio	-	-	−1.99
Trunk ipsilateral flexion, deg	-	-	0.10
Discrimination			
Apparent performance AUC	0.80 (95% CI 0.68, 0.91)	0.67 (95% CI 0.49-0.82)	0.76 (95% CI 0.65, 0.87)
Optimism-corrected performance AUC	0.78 (95% CI 0.66, 0.90)	-	0.72 (95% CI 0.61, 0.82)
Calibration			
Intercept	0.00 (95% CI −0.54, 0.54)	−0.79 (95% CI −1.44, −0.14)	-0.00 (95% CI -0.41, 0.41)
Slope	1.22 (95% CI 0.68, 1.77)	0.53 (95% CI −0.09, 1.14)	1.38 (95% CI 0.85, 1.92)
Overall explained variation, *R*^ 2^	0.18 (95% CI 0.10, 0.25)	0.03 (95% CI 0.00, 0.06)	0.15 (95% CI 0.10, 0.20)

ACL, anterior cruciate ligament; AUC, area under the curve; CMJ, countermovement jump.

### Updated Model

**Figure 3.**
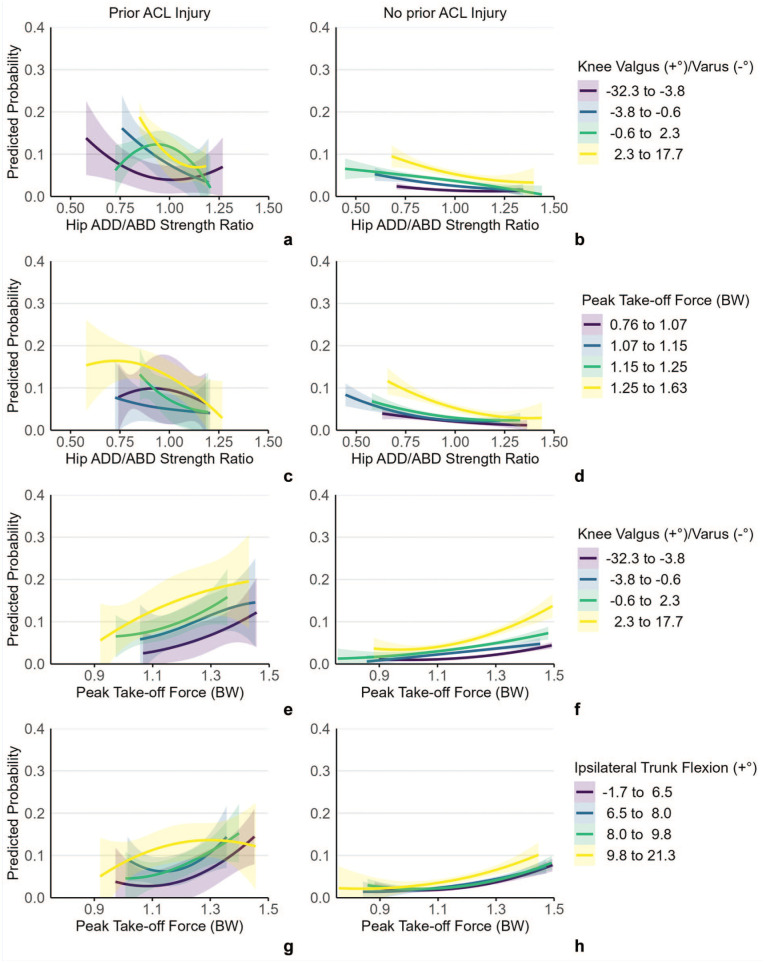
Interaction plots showing the predicted probabilities of ACL injury for different combinations of hip ADD/ABD strength ratio, peak take-off force, knee valgus/varus, and ipsilateral trunk flexion in players with and without previous ACL injury included in the updated multivariable prediction model. ABD, abduction; ACL, anterior cruciate ligament; ADD, adduction.

The model was redeveloped with the inclusion of hip adductor/abductor strength ratio, trunk ipsilateral flexion, and re-estimation of coefficients, resulting in increased apparent (AUC, 0.76; 95% CI, 0.66, 0.86) and optimism-corrected (AUC, 0.72; 95% CI, 0.61, 0.82) performances in the combined cohort (Table 3). Calibration of the updated model resulted in an intercept of –0.00 and slope of 1.38 (Figure 2). Predicted probabilities of ACL injury varied for different combinations of dynamic knee valgus/varus, ipsilateral trunk flexion, peak take-off force, hip adductor/abductor strength ratio, and previous ACL injury (Figure 3). Of the 642 players in the combined cohort, 125 players had missing data for ≥1 predictor included in the multivariable model. Complete-case analysis demonstrated similar model performance to multiple imputation, with an apparent AUC of 0.75 (95% CI, 0.64, 0.85), a calibration slope of 1.81 (95% CI, 1.00, 2.62), and an intercept of –0.00 (95% CI, –0.42, 0.42). The characteristics of athletes with missing data were similar to complete cases (Supplementary Table 1). Missing data for different datasets are presented in Supplementary Figure 1. A web-based risk calculator is available at: https://aclrisk.shinyapps.io/ACL_Injury_Risk/

## Discussion

This is the first study to externally validate and update a field-based clinical prediction model for ACL injury risk in woman/girl footballers. The main findings were that: (1) the original model demonstrated poor discrimination (AUC, 67%) and miscalibrated risk estimates in the external validation cohort; and (2) the updated model including previous ACL injury, dynamic knee valgus, peak take-off force, hip adduction/abduction strength ratio, and ipsilateral trunk flexion achieved possibly helpful optimism-corrected discrimination (AUC, 72%) but lacked precision in risk estimates. In practical terms, the model may help identify groups of players at higher risk, but is not yet able to accurately predict ACL injury risk for individual players.

Several reasons could explain why the original model performed poorly and overestimated risks in the validation cohort. First, the validation cohort had a lower ACL injury incidence than the development cohort, which can lead to systematic overestimation of risks.^46^ Second, the strength of the association between predictors (e.g., previous ACL injury and dynamic knee valgus) and injury differed between cohorts, potentially reflecting temporal changes in training environments, injury prevention practices, or player characteristics.^3^ Although the validation and development cohorts were extracted from similar populations, data collection occurred 1 to 2 years apart. In that time, factors such as improvement in facilities, increased staff numbers, and greater professionalization of players could have changed the risk profile of the players, such as contributing to a lower reinjury proportion in the validation cohort compared with the development cohort.

To address these limitations, we combined the validation and development cohorts and included hip adductor/abductor strength ratio and ipsilateral trunk flexion angle into an updated model. The performance of this model is comparable with clinical prediction models in other domains, including hip arthroscopy recovery (AUC, 0.75) ^26^, neck pain (AUC, 0.73),^51^ low back pain (AUC, 0.62-0.75), ^25^ and heart disease (AUC, 0.72).^1,15^ However, calibration remained suboptimal, particularly at higher predicted risk levels. Although the model was accurate for low injury probabilities (0 to 10%), it consistently underestimated risk at higher ranges. This calibration instability indicated that predictions for individual players could vary significantly; for example, a player with a predicted risk of 10% could have an actual risk anywhere between 5% and 70%.

The updated model includes previous ACL injury, dynamic knee valgus and lateral trunk flexion during a triple hop, peak take-off force during a CMJ, and hip adductor-to-abductor strength ratio. Previous ACL injury is the most consistently identifed risk factor for ACL reinjury in women.^11^ Increased injury risk might occur because rehabilitation fails to address the original caustive risk factors,^43^ or may reflect structural graft changes,^36^ or persistent neuromuscular deficits that occur as a consequence of injury.^34^ Peak take-off force is the highest force during the jump phase of the CMJ, and greater take-off forces during CMJs may reflect higher quadriceps forces, which increase anterior shear forces and ACL strain.^32^ Further, higher dynamic knee valgus and ipsilateral trunk flexion are common mechanisms for ACL injuries in women,^24^ as they shift the GRF vector lateral to the knee,^38^ increasing knee abduction moments and ACL loading.^4,12,38^ Finally, a low hip adductor/abductor ratio may indicate weaker adductors relative to abductors, impairing the ability to generate knee varus moments to counteract valgus forces during high-risk tasks.^33^ However, the casual relationship of this variable requires further investigation.

## Limitations

Our study has limitations. The relatively low number of injuries limited model calibration and the stability and precision of predictor effect estimates, and larger datasets are required to develop more stable individual-level risk prediction models. Although missing data were low, some predictors had incomplete observations which may impact model robustness. Moreover, our testing battery was limited to strength and biomechanical factors and we did not examine potential nonlinear interactions between risk factors, which may have limited model accuracy given the dynamic and multifactorial nature of ACL injury risk.^5^ Finally, strength and biomechanical measures were also assessed at a single preseason timepoint, and potential changes over the study period were not captured.

### Clinical Implications

The updated prediction model employs measures that can be collected in <10-minutes per player via comercially available equipment and questionnaires, making it feasible for use in team sport settings. Importantly, 4 of the 5 predictors in the updated model are modifiable, highlighting the potential for targeted injury prevention strategies if their causal role is confirmed.

### Future Directions

The updated model requires further external validation in larger cohorts to assess stability and generalizability,^35^ including other populations (e.g., men’s football, other sports and countries). In addition, future research should evaluate the model’s clinical utility using net benefit analysis to determine if it offers value beyond conventional strategies, such as treating all or no players.^49^

## Conclusion

The original prediction model did not generalize well to an independent cohort of footballers, showing poor discrimination and risk overestimation. The updated model demonstrated possibly useful discriminatory ability but limited calibration and instability. While it may support group-level risk stratification, it is not yet suitable for estimating individual player risk.

## Supplemental Material

sj-docx-1-sph-10.1177_19417381261457613 – Supplemental material for Can Field-Based Screening Predict ACL Injury Risk in Women Footballers? External Validation of a Prediction Model Supplemental material, sj-docx-1-sph-10.1177_19417381261457613 for Can Field-Based Screening Predict ACL Injury Risk in Women Footballers? External Validation of a Prediction Model by Yuri Lopes Lima, Tyler Collings, Michelle Hall, Laura E. Diamond and Matthew N. Bourne in Sports Health
