# Supplementary material for: Can Field-Based Screening Predict ACL Injury Risk in Women Footballers? External Validation of a Prediction Model
Source: Sports Health. 2026 Jul 23:19417381261457613. Online ahead of print. doi: 10.1177/19417381261457613 (PMC13400702; doi:10.1177/19417381261457613)
Supplement: sj-docx-1-sph-10.1177_19417381261457613 – Supplemental material for Can Field-Based Screening Predict ACL Injury Risk in Women Footballers? External Validation of a Prediction Model [file sj-docx-1-sph-10.1177_19417381261457613.docx]

**SUPPLEMENTARY MATERIAL**

Supplementary Material 1. Injury report form

**
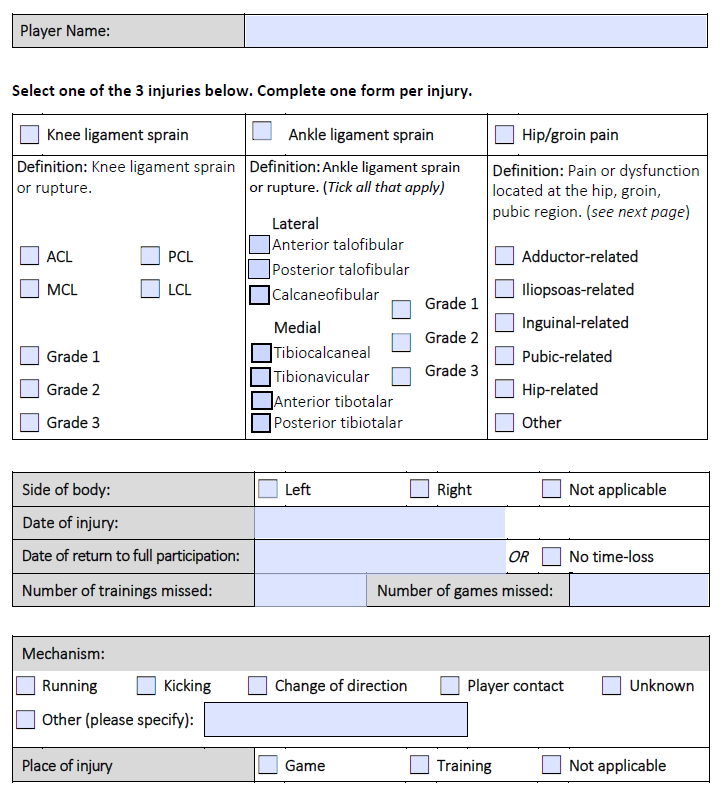
**

**
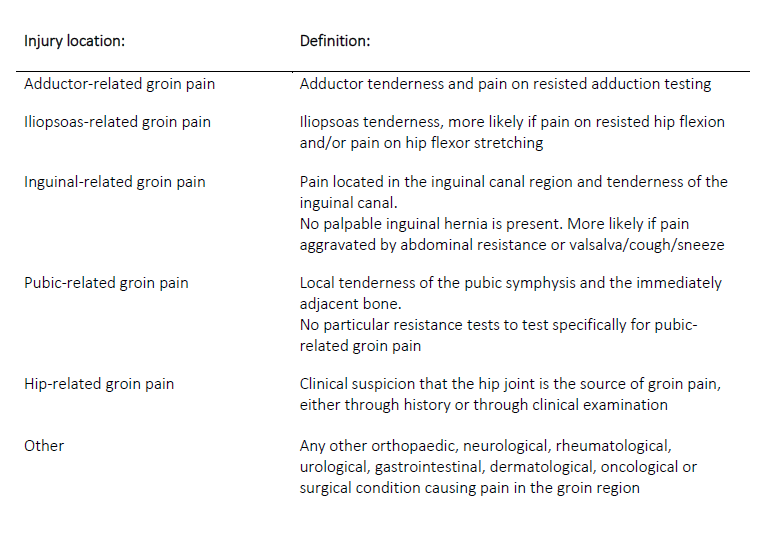
**

Supplementary Material 2. Injury history questionnaire

**Injury History Questionnaire**

| **First Name** |  | | **Date of birth** | | |  | | | | | |
| --- | --- | --- | --- | --- | --- | --- | --- | --- | --- | --- | --- |
| **Last Name** |  | | **Today’s date** | | |  | | | | | |
| **Height (m)** |  | | **Kicking foot** | | | 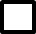 | | Left | | 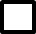 | Right |
| Are you (or suspect you may be) pregnant? | | | | | | 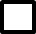 | | Yes | | 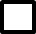 | No |
| Are you currently taking any form of contraceptive medication? | | | | | | 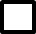 | | Yes | | 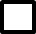 | No |
| **Have you ever ruptured an ACL?** | | | 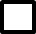 No | | | 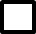 | | Left | | 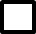 | Right |
| **If yes, what date was the most recent ACL injury? / / (dd/mm/yyyy)** | | | | | | | | | | | |
| **What tendon graft was used?** | | 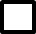Hamstrings | | 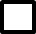 | Patella | | 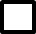 | | Quadriceps | | |
| **Did you have ACL reconstruction surgery?** | | | 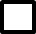 No | | | 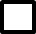 | | Yes | |  |  |
| **If yes, what date was the surgery? / / (dd/mm/yyyy)** | | | | | | | | | | | |

| **INSTRUCTIONS:** Please tick any of the injuries listed below that you have experienced in the **last 12 months** due to **football related activities**. Please also tick whether the injury caused you to **miss one or more games or training** sessions (not including the day the injury happened). | | | | | | | | | |
| --- | --- | --- | --- | --- | --- | --- | --- | --- | --- |
|  | **Leg** | | | | **Game/training missed?** | | | | |
| Ankle ligament sprain |  | Left |  | Right | |  | Yes |  | No |
| Calf strain |  | Left |  | Right | |  | Yes |  | No |
| Achilles tendinopathy |  | Left |  | Right | |  | Yes |  | No |
| Knee ligament sprain |  | Left |  | Right | |  | Yes |  | No |
| Patellar tendinopathy |  | Left |  | Right | |  | Yes |  | No |
| Hamstring strain |  | Left |  | Right | |  | Yes |  | No |
| Quadriceps strain |  | Left |  | Right | |  | Yes |  | No |
| Hip or groin pain |  | Left |  | Right | |  | Yes |  | No |

**Risk-Taking Behaviour Scale**

| When practising my sport I have sometimes been involved in accidents that are caused by my somewhat irresponsible attitude. |  | Strongly agree |  | Agree |  | Neutral |  | Disagree |  | Strongly disagree |
| --- | --- | --- | --- | --- | --- | --- | --- | --- | --- | --- |
| I think I am very careful and far-sighted when I practice my sport. |  | Strongly agree |  | Agree |  | Neutral |  | Disagree |  | Strongly disagree |
| My friends or colleagues who are experts in the activity think I take too many risks when I practise my sport. |  | Strongly agree |  | Agree |  | Neutral |  | Disagree |  | Strongly disagree |

| **Ankle Injury History** | | | | | | | | | | | | | | | |
| --- | --- | --- | --- | --- | --- | --- | --- | --- | --- | --- | --- | --- | --- | --- | --- |
| Have you ever sprained an ankle? | | | |  | | | | No | |  | | Left | |  | Right |
| How long ago was your most recent ankle sprain? | | | | | | | | | | | | | | | |
|  | 0-6 months |  | 6-12 months |  | | | | | 1-2 years | | | | |  | 2 years or more |
| How many times have you sprained the same ankle in your life? | | | | | | 1 2 3 4 5 6 7 8 9 10 10+ | | | | | | | | | |
| Does this ankle ever feel like ‘giving way’ or feel unstable? | | | | |  | | Yes | | | |  | | No | | |
| How often have you experienced a feeling of ‘giving way’ in the last 6 months? | | | | | 1 2 3 4 5 6 7 8 9 10 10+ | | | | | | | | | | |

Supplementary Figure 1. Missing data by dataset.


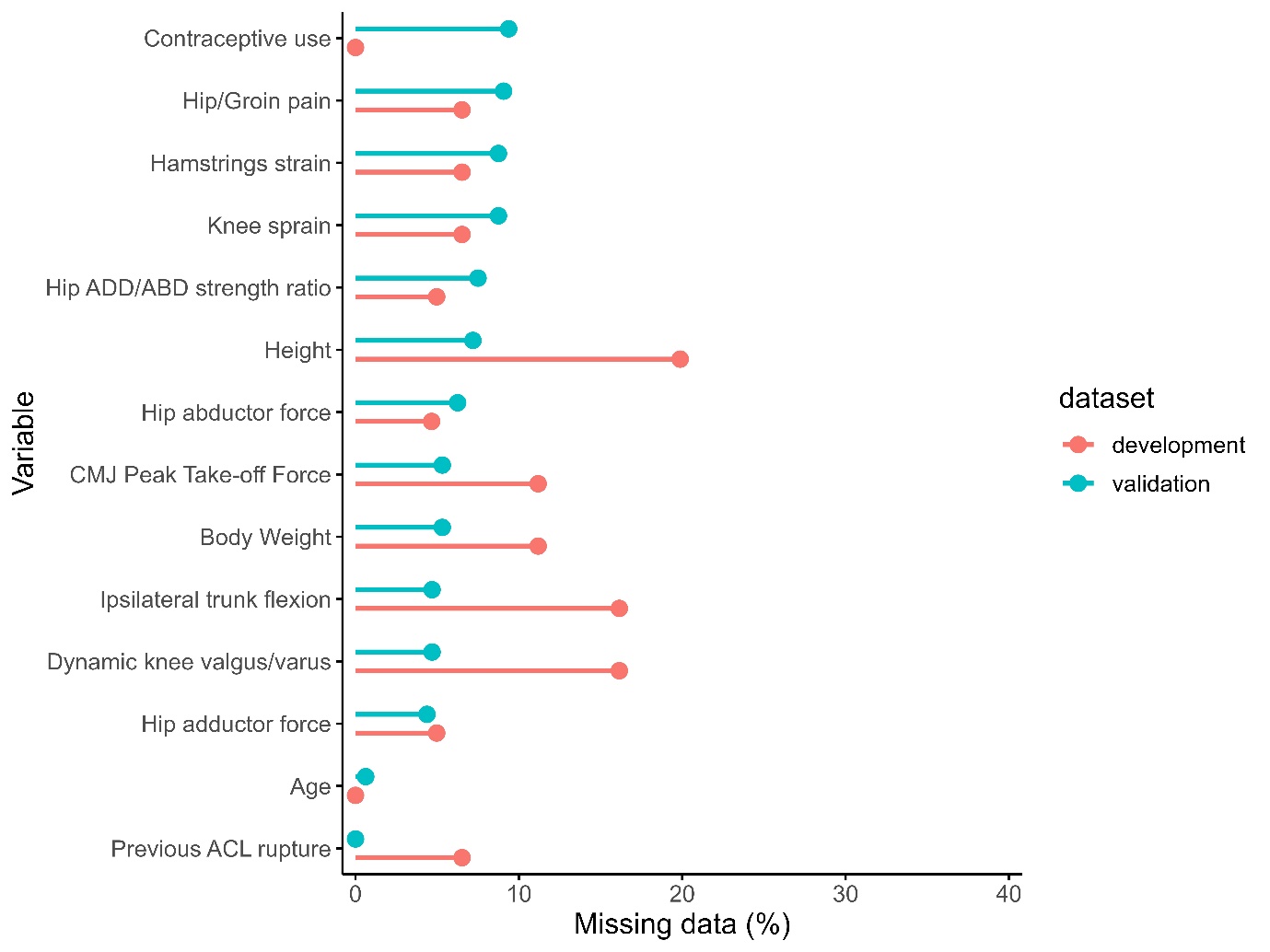


Supplementary Table 1. Demographics and predictors of players with missing data and complete cases.

|  | Complete cases (n=517) | Players with missing data (n=125) |
| --- | --- | --- |
| Age (years)^a^ | 21 (9) | 22 (8) |
| Height (m) | 1.73 (0.08) | 1.74 (0.08) |
| Body weight (kg) | 66.3 (9.5) | 66.2 (10.2) |
| Contraceptive use, *n*(%)^b^ | 116 (23.7%) | 21 (17.0%) |
| Previous ACL rupture, *n*(%)^b^ | 48 (9.3%) | 9 (8.6%) |
| Previous knee sprain, *n*(%)^b^ | 37 (7.5%) | 8 (7.8%) |
| Previous hamstring strain, *n*(%)^b^ | 55 (11.2%) | 13 (12.7%) |
| Previous hip and groin pain, *n*(%)^b^ | 69 (14%) | 15 (14.7%) |
| CMJ peak take-off force (BW) | 1.16 (0.13) | 1.16 (0.15) |
| Maximum isometric hip abductor force (N) | 141 (25) | 145 (27) |
| Maximum isometric hip adductor force (N) | 139 (29) | 140 (31) |
| Hip adductor/abductor strength ratio | 0.99 (0.15) | 1.00 (0.16) |
| Dynamic knee valgus (+°)/varus (−°) | -1.2 (5.6) | -0.4 (5.9) |
| Ipsilateral trunk flexion (°) | 8.3 (2.9) | 7.9 (3.09) |
